# Metastatic Osteoarticular Infective Endocarditis by Methicillin-sensitive Staphylococcus Aureus

**DOI:** 10.7759/cureus.8124

**Published:** 2020-05-14

**Authors:** Asseel Al-bayati, Abbas Alshami, Mohammed AlAzzawi, Alsadiq Al Hillan, Mohammad Hossain

**Affiliations:** 1 Internal Medicine, Jersey Shore University Medical Center, Neptune City, USA; 2 Internal Medicine, University of Baghdad College of Medicine, Baghdad, IRQ; 3 Internal Medicine, Hackensack Meridian Health, Jersey Shore University Medical Center, Neptune City, USA; 4 Internal Medicine, Dorrington Medical Associates, Houston, USA

**Keywords:** infective endocarditis, methicillin-sensitive staphylococcus aureus, paronychia, septic emboli, skin infection

## Abstract

Infective endocarditis (IE) is a well-known complication of bacteremia with high-risk microorganisms such as *Staphylococcus* and *Streptococcus*. Skin and soft tissue infections with Staphylococcus remain a significant cause of bacteremia and IE, even with proper prompt management of the source of infection and the absence of risk factors. Although methicillin-resistant *Staphylococcus aureus* is a well-known etiology for osteoarticular septic emboli in IE, healthcare providers should be aware of the hidden virulence of methicillin-sensitive *Staphylococcus aureus* for metastatic osteoarticular infection. We report a case of IE with septic vertebral embolic lesion complicating a properly managed acute paronychia.

## Introduction

Infective endocarditis (IE) is an infection of the endocardial linings of the heart, with the involvement of one or more of the heart valves (native or artificial). IE is associated with a broad array of complications, and many factors play a role in the development of those complications. One of the many complications is metastatic infection, which is a form of septic embolization [1,2].

The prevalence of musculoskeletal pyogenic metastasis is considered low. Furthermore, the characteristics of this manifestation are not well known yet. Osteoarticular symptoms in IE can be related to immunological disorders associated with IE or they can be related to bacterial embolization. In IE among non-drug users, *Streptococcal* infection was highlighted to be the etiology for metastatic osteoarticular infections (OAIs).

In this case report, we emphasize the role of methicillin-sensitive *Staphylococcus aureus* (MSSA) in complicated IE with vertebral osteomyelitis, and the importance of early transesophageal echocardiography in OAIs (septic arthritis and vertebral osteomyelitis) to detect IE and guide the treatment as early as possible to achieve the best outcome and low recurrence.

## Case presentation

A 64-year-old man presented with progressive neck pain for three weeks. The patient endorsed that the pain was mild initially, radiating to the right shoulder, and aggravated by neck movement. He reported some relief with non-steroidal anti-inflammatory medications at home. He presented to the emergency department as the pain reached intolerable levels, not responding to pain-relieving medications anymore. The patient denied numbness, weakness, neck trauma, morning stiffness, limitation of movement, abdominal pain, or change appetite. Moreover, there was no report of fever, chills, fatigue, shortness of breath, palpitation, headache, vision changes, or seizures. His medical history was only significant of recent acute complex paronychia, which was surgically drained and treated with a seven-day course of cephalexin 500 mg twice daily. The paronychia resolved completely per the patient. There was no history of chronic use of medications.

On physical examination, his blood pressure was 115/82 mm Hg, heart rate was 81 beats/minute, respiratory rate was 15 breaths per minute, temperature was 98.2°F, and pulse oxygenation was 98% on room air. Neck examination was remarkable for mid-line point tenderness over the cervical vertebrae C6 and C7, with intact neurological signs.

**Figure 1:**
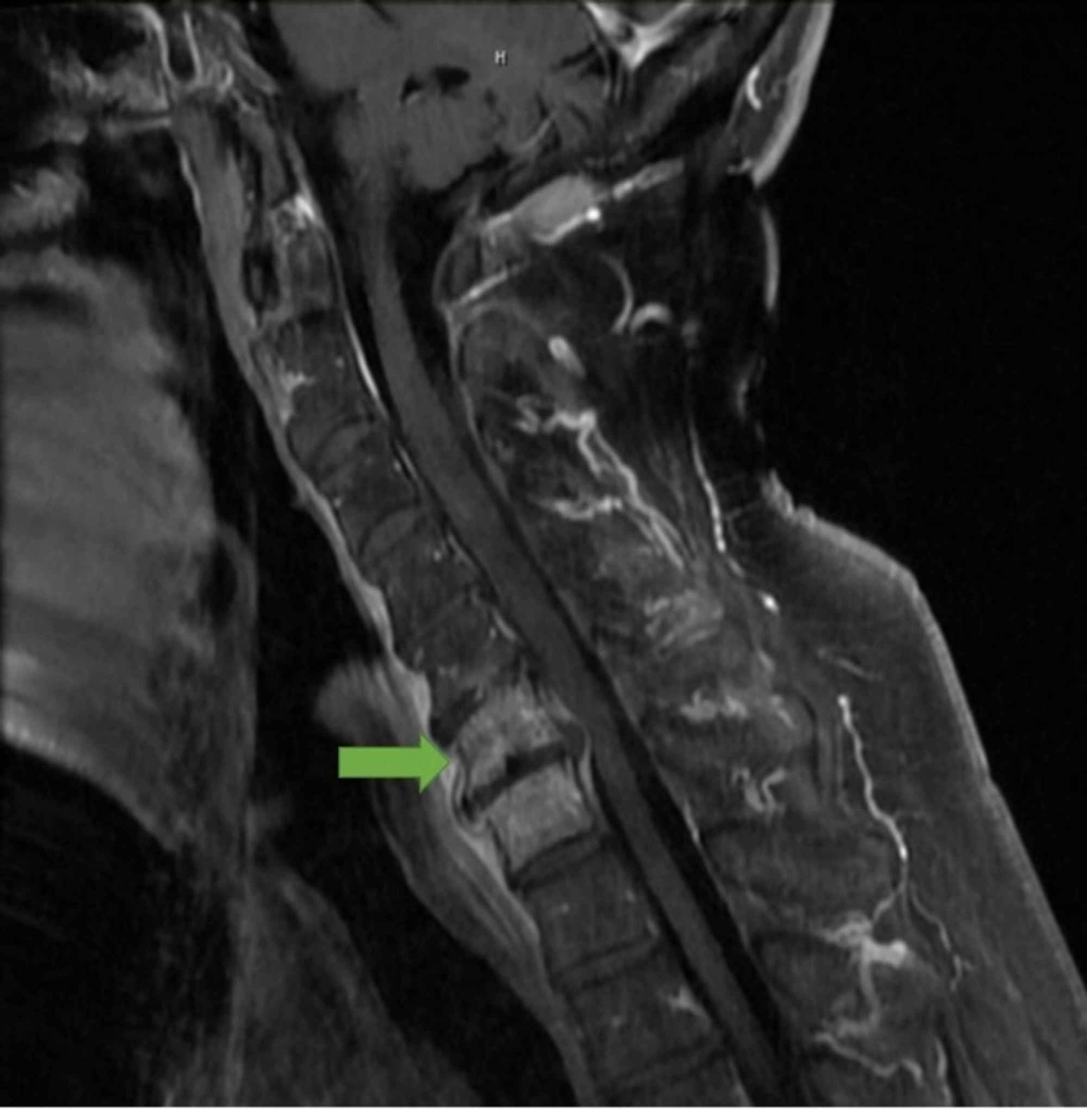
MRI of the spine with intravenous contrast showing cervical spine C6-C7 discitis/osteomyelitis

**Figure 2:**
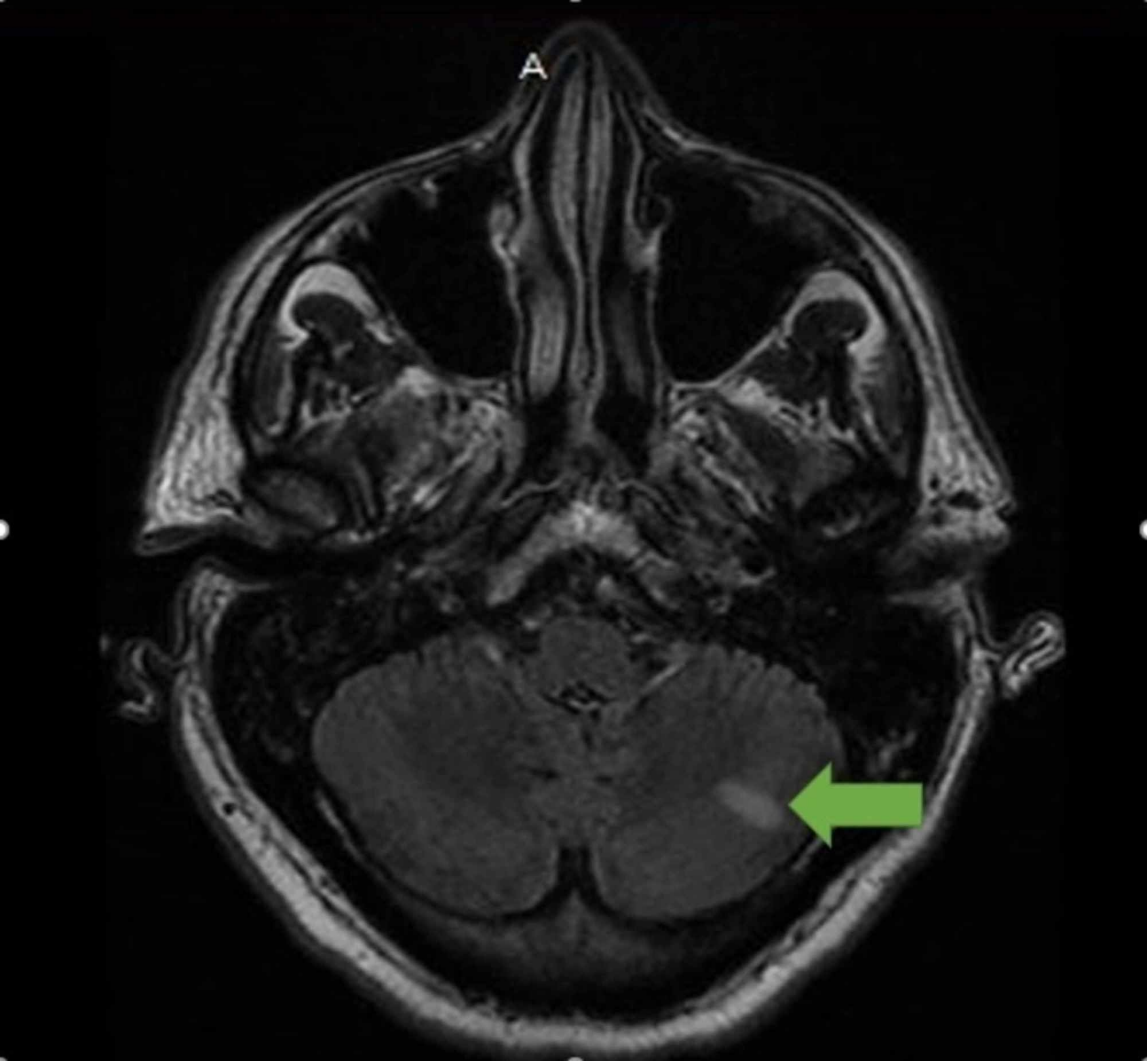
MRI of the brain showing embolic phenomena at the left cerebellum

**Figure 3:**
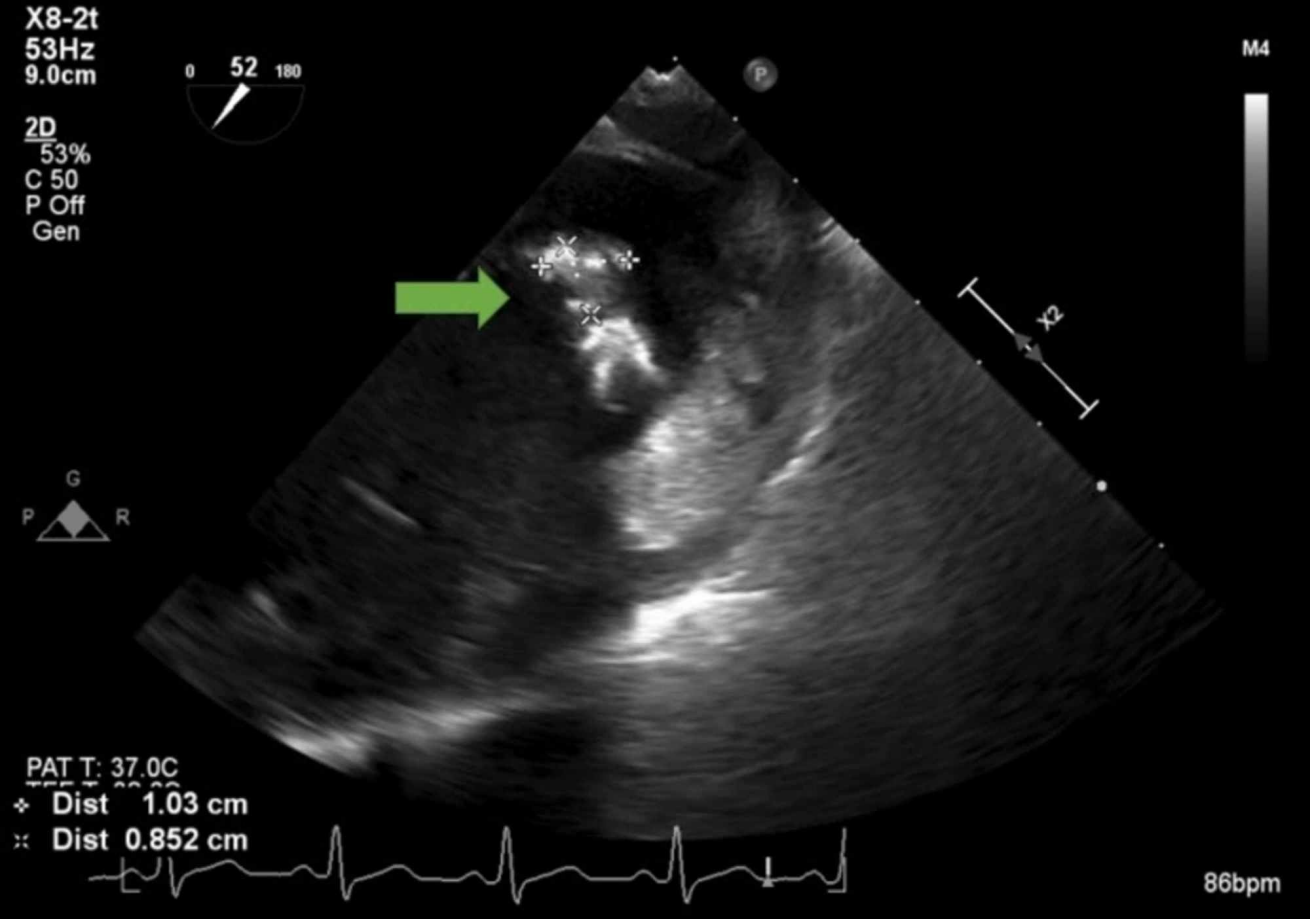
Transesophageal echocardiography showing mitral valve vegetation measuring 1 cm x 1 cm

Initial laboratory workup results (Table 1) were significant for a white blood cell count of 17.4 × 10^3^/uL (reference range: 4.5 to 11 x 10^3^/µL). A magnetic resonance imaging (MRI) scan of the cervical spine revealed a decreased T1 marrow signal of the C6 and C7 vertebral bodies with associated diffuse edema and enhancement, suggestive of osteomyelitis (Figure 1). The patient was admitted for osteomyelitis and was started empirically on vancomycin 2 g intravenously dosed at 1 g every 12 hours for two days. The following day, the patient suddenly developed slurred speech and behavioral changes. A brain MRI revealed foci of restricted diffusion and increased T2 signal intensity in the left cerebellum and the left parieto-occipital cortex most consistent with embolic infarcts (Figure 2). Two blood cultures obtained on admission were positive for MSSA, which was sensitive to cephalosporins. Two-dimensional transthoracic echocardiography showed a moderate mobile mass consistent with possible vegetation on the mitral valve, and subsequent transesophageal echocardiography showed vegetation on the atrial side of the posterior leaflet of the mitral valve with calcifications (Figure 3).

The antibiotics were narrowed to nafcillin 3 g every six hours for six weeks. Subsequent blood cultures came back negative, and the patient was discharged from the hospital in stable condition. The patient was sent to a rehabilitation facility, and he was doing well on two-week follow-up after discharge from the hospital.

**Table 1:** Laboratory workup

	Result	Reference Range
White blood cell count	17.4 K/uL	4.5–11 K/uL
Hemoglobin	15.1 g/dL	12–16 g/dL
Hematocrit	44.1%	35–48%
Platelet count	453 K/uL	140–450 K/uL
Blood urea nitrogen	11 mg/dL	5–25 mg/dL
Creatinine	0.8 mg/dL	0.44–1 mg/dL

## Discussion

IE is a life-threatening disease and may be acquired in the community or through healthcare exposure [3]. Risk factors include age older than 60 years, male sex, dental diseases, intravenous drug abuse (IVDA), and structural heart disease. More than half of all IE cases in the United States and Europe occur in patients older than 60 years [4]. Due to the lower incidence of rheumatic heart disease and increased survival age, the median age of patients with IE has increased over the past 40 years [4-6]. Additionally, men are 90% more likely to develop IE than women [4,7,8]. Another known risk factor for IE is structural heart disease (e.g., rheumatic heart disease). However, IE can develop in patients who have no history of structural heart disease (in nearly one in four cases) [9]. A Spanish study reported an increased proportion of IE in patients with undocumented structural heart disease from 2001 to 2013 (compared with 1987 to 2001) [8]. Such patients were more likely to be immunosuppressed and acquired the infection after exposure to healthcare settings [9-11].

The three most common causes of IE worldwide are *Staphylococci*, *Streptococci*, and *Enterococci*. In the United States and most developed countries, *Staphylococcus aureus* is the most common cause of IE and is a common cause of healthcare-associated IE [12].

MSSA patients had significantly more unknown origin of bacteremia and experienced a significantly higher rate of major embolism than methicillin-resistant *Staphylococcus aureus* (MRSA) patients [4]. According to Chang et al., patients with endocarditis due to MRSA were significantly more likely to experience persistent bacteremia compared with patients with MSSA endocarditis [13].

Yoon et al. reported that if persistent bacteremia is documented, there should be a high likelihood of MRSA endocarditis and prolonged anti-*Staphylococcus* therapy or a more potent management must be implemented. This was based on the higher mortality for MRSA IE (50.0%) than for MSSA IE (9.1%) (p=0.019) [11]. However, for our patient, the contrary was true. He presented with a history of persistent bacteremia of MSSA that resulted in left-sided MSSA endocarditis complicated by metastatic infection despite proper treatment course.

What is interesting about the presented case is that the MSSA bacteremia originated from the skin in an immunocompetent 61-year-old man with no history of comorbidity or structural heart disease, IVDA, or cardiac devices. Such presentation is commonly seen in MRSA IE with high comorbidity and right-sided vegetation, nosocomial infection, persistent bacteremia, mechanical ventilation, prior antibiotic use, and a higher rate of mortality (p<0.05) [14].

Our patient was being treated for complicated IE, which was defined based on the two major modified Duke's criteria (two separate positive blood cultures with microorganisms suggestive of IE, and evidence of endocardial involvement confirmed by echocardiogram). The timeline between the acute paronychia and subsequent complications with the absence of other sources of infection suggests the site of paronychia as the port of entry of the MSSA to the blood. The MSSA here acted unexpectedly, leading to persistent bacteremia in an immunocompetent individual who finished the proper course of antibiotics and extended to the point of vegetating the left side of the heart to present as septic embolization to the vertebrae and brain. The absence of any risk factors to develop IE, such as a prosthetic or damaged heart valve, and the proper management of the paronychia with surgical drainage and appropriate antibiotics did not prevent the seeding of the bacteria into the mitral valve and the vertebrae, and the resultant IE and osteomyelitis.

OAIs associated with IE were seen in 77% of axial skeleton and 20% of vertebral osteomyelitis. The microorganisms detected usually predict a particular type of pyogenic OAI. For instance, Murillo et al. in an observational study (1993-2014) published in 2018 in Barcelona, Spain, has shown that vertebral osteomyelitis is 2 to 8 times more likely to be caused by “less virulent” bacteria (*Streptococcus* viridans, *Streptococcus* bovis, *Enterococcus*, and coagulase-negative *Staphylococcus aureus*) [15-18]. *Staphylococcus* aureus was reported more commonly in axial skeleton septic metastasis.

The prevalence of musculoskeletal pyogenic metastasis is considered low. Furthermore, the characteristics of this manifestation are not well known yet. The presented case exceptionally shows the affinity of MSSA for vertebral osteomyelitis.

## Conclusions

Skin and soft tissue infections with MSSA remain a significant cause of persistent bacteremia and IE, even with proper management and an absence of risk factors. Healthcare providers should be aware of the potential virulence of MSSA IE to be persistent. A re-evaluation, optimization of an antimicrobial regimen, the choice of antibiotic, the duration of therapy, and early surgical intervention may be required.

Our case reports an unusual complication of MSSA persistent bacteremia that resulted in complicated IE., which is a disease behavior frequently seen in MRSA. The importance of ascertaining full eradication of MSSA skin infections with vigilant recognition of signs of bacteremia is of utmost importance to avoid IE complications. Moreover, the presented case, non-classically, shows the affinity of MSSA for vertebral osteomyelitis for reasons not well understood. Practically, transesophageal echocardiography should be habitually considered in patients with OAIs (axial and vertebral).
